# Metagenomic identification of a new sarbecovirus from horseshoe bats in Europe

**DOI:** 10.1038/s41598-021-94011-z

**Published:** 2021-07-19

**Authors:** Jack M. Crook, Ivana Murphy, Daniel P. Carter, Steven T. Pullan, Miles Carroll, Richard Vipond, Andrew A. Cunningham, Diana Bell

**Affiliations:** 1 National Infection Service, Public Health England, Porton Down, Salisbury, UK; 2 Centre for Ecology, Evolution and Conservation, School of Biological Sciences, University of East Anglia, Norwich, NR4 7TJ UK; 3 Wellcome Trust Centre for Human Genetics, Nuffield Department of Medicine, Oxford University, Oxford, UK; 4 Institute of Zoology, Zoological Society of London, London, NW1 4RY UK; 5 NIHR Health Protection Unit in Emerging and Zoonotic Infections, Department of Clinical Infection, Microbiology and Immunology, University of Liverpool, Liverpool, L69 7TX UK

**Keywords:** Viral reservoirs, SARS-CoV-2

## Abstract

The source of the COVID-19 pandemic is unknown, but the natural host of the progenitor sarbecovirus is thought to be Asian horseshoe (rhinolophid) bats. We identified and sequenced a novel sarbecovirus (RhGB01) from a British horseshoe bat, at the western extreme of the rhinolophid range. Our results extend both the geographic and species ranges of sarbecoviruses and suggest their presence throughout the horseshoe bat distribution. Within the spike protein receptor binding domain, but excluding the receptor binding motif, RhGB01 has a 77% (SARS-CoV-2) and 81% (SARS-CoV) amino acid homology. While apparently lacking hACE2 binding ability, and hence unlikely to be zoonotic without mutation, RhGB01 presents opportunity for SARS-CoV-2 and other sarbecovirus homologous recombination. Our findings highlight that the natural distribution of sarbecoviruses and opportunities for recombination through intermediate host co-infection are underestimated. Preventing transmission of SARS-CoV-2 to bats is critical with the current global mass vaccination campaign against this virus.

## Introduction

The sources of the current COVID-19 pandemic and of the 2003 Severe Acute Respiratory Syndrome (SARS) epidemic are unknown^1^. Currently, the natural hosts of both SARS-CoV and SARS-CoV-2 (family Coronaviridae; subgenus *Sarbecovirus*)^2^, the causative agents of SARS and COVID-19 respectively, are thought to be horseshoe bats (Rhinolophidae), with the zoonotic spillover process involving one or more intermediate hosts, during which time viral mutation, recombination and/or amplification could have occurred^3–7^. Phylogenetic analyses of novel horseshoe bat sarbecoviruses in China have shown these to be most closely related to both SARS-CoV and to SARS-CoV-2^3,4,7^. Recently, a SARS-CoV-2-like virus was also reported from a species of horseshoe bat in Thailand^8^.

The range of horseshoe bats extends across much of the Old World, but most sampling for coronaviruses has been conducted in East and South East Asia, where around 50 SARS-related coronaviruses (SARSr-CoVs) have been detected across ten species of bat, with 48 of these being from nine species of horseshoe bat^8–11^. Here we expand the investigation of SARSr-CoVs to the lesser horseshoe bat (*Rhinolophus hipposideros*) in the UK, which is at the western extreme of the range of the Rhinolophidae.

## Methods

### Sample collection

Faecal samples were collected from lesser horseshoe bats *R. hipposideros* during routine annual population monitoring surveys at three sites in August and September 2020 following approval by the University of East Anglia Ethics Committee and adhering to UK Government COVID-19 safety regulations in place at the time. Samples were either obtained from individual bats at the time of capture or from under-roost sampling. In Somerset (n = 20 bats) and Monmouthshire, Wales (n = 7) bats were captured using harp traps or mist nets placed near roosts or in woodland under government license. A faecal pellet was collected from each of 23 bats held individually in sterile holding bags and the other four samples were collected as anal swabs using rayon-tipped dry swabs (MW100; Medical Wire & Equipment), which were taken from bats that did not defaecate when captured. All bats were released at the site of capture immediately after sample collection. In Gloucestershire, 26 faecal pellets were collected by placing a sterile sheet underneath a lesser horseshoe roost and any droppings that landed directly onto the sheet within 30 min were collected. Each faecal sample was placed into an individual sterile tube containing 2 ml RNAlater, refrigerated overnight and stored frozen prior to analysis.

### Genomic sequencing

For metagenomic analysis, samples were homogenised by vortexing and spiked with 10^6 genome copies per ml of Hazara virus as an internal control. A 140 µl aliquot of each sample was extracted using the QIAamp Viral RNA extraction kit. Extracts were DNase treated, reverse transcribed and randomly amplified using a Sequence-Independent Single-Primer Amplification (SISPA) based method described in detail previously^12^. Illumina sequencing used the Nextera XT protocol with 2 × 150-bp paired-end sequencing on a MiSeq. Nanopore library preparation was as described previously^12^ and sequencing was performed on an Oxford nanopore GridION with base calling via Guppy. Nanopore reads were trimmed using NanoFilt^13^ to remove 25 bp SISPA primer sequences from the start and end of each read. Raw data and the assembly sequence are deposited at NCBI under BioProject PRNJA706167.

### Genomic analyses

Read-level taxonomic classification for each sample was performed using Kraken2 against the RefSeq database (2.0.8-beta)^14^. *De novo* genome assembly was performed using SPAdes (3.15.1) for both Illumina and Illumina/nanopore hybrid assemblies^15^. Contigs of interest were identified using BLASTn^16^. Illumina reads were mapped to the assembled contigs of interest using BWA-MEM^17^ and nanopore reads using Minimap2^18^. Read depth values were generated using SAMtools (1.10)^19^.

The assembled genome was aligned with selected reference genomes (NC_014470.1, KJ473814.1, NC_045512.2) in MEGA X (10.2.4) using MUSCLE alignment^20,21^. Nucleotide and codon alignments were generated for each gene with visual depictions of alignment and pairwise alignment scores generated in JALVIEW (2.11.1.3)^22^.

Aligned nucleotide sequences from 21 sarbecoviruses obtained from GenBank were used to generate maximum likelihood trees using IQTREE (2.0.3)^23^. The best fitting nucleotide substitution model was used as selected by ModelFinder for each individual phylogeny^24^. Nodes were evaluated using UFBoot with 1,000 bootstrap approximations^25^. Phylogenetic tree visualisation was carried out in FigTree (5.7)^26^.

### Homology modelling

SWISS-MODEL was used for homology modelling of the tertiary structure of the receptor binding domain (RBD) in RhGB01^27^. The input target sequence was residues 324–515 in the spike protein sequence. SWISS-MODEL performs a template search of BLAST and HHblits databases for pre-existing characterised structures with homology. The most suitable template was selected according to the highest Global Model Quality Estimate (GMQE) and lowest Quaternary Structure Quality Estimate (QMEAN). GMQE expresses the accuracy of the model built as a number between 0 and 1. QMEAN uses statistical potentials of mean force to generate global and per residue estimates. Modelling of the complete S protein amino acid sequence did not yield reliable models (GMQE < 0.65), whereas modelling of the RBD amino acid sequence yielded a reliable model (GMQE > 0.7, QMEAN − 2.18). The template structure selected was the crystal structure of SARS-CoV RBD complexed with a neutralising antibody (PDB ID 2DD8).

The predicted 3D structure was visualised in Visual Molecular Dynamics^28^ using sequence alignment and STAMP structural alignments for superimpositions. Structural identity is represented by a Qres (Q) score where Q is measure of structural similarity measuring the fraction of Cα atoms that superimpose. Validation of the homology model was performed using ProCheck^29,30^ and ERRAT2^31^ on the structural analysis and verification server (SAVES). ProCheck generates a Ramachandran plot which classifies the torsion angles of each residue in the predicted 3D structure into 3 groups to assess its validity. ERRAT2 uses a database of 96 high-resolution protein structures to classify the 6 types of atomic interactions (carbon–carbon, carbon–nitrogen, carbon–oxygen, nitrogen-nitrogen, nitrogen–oxygen, oxygen–oxygen) into correct or incorrectly determined protein regions based on the atomic interactions. This provides an overall quality factor for non-bonded atomic interactions expressed as the percentage of a protein for which the calculated values sits below the set rejection limits, with a score > 50 indicating a high-quality model^31^.

## Results

### Metagenomic analysis revealed an unclassified betacoronavirus in a single sample with genome organisation consistent with *Sarbecovirus*

An initial screening of the 53 samples identified one sample from Gloucestershire with > 650 reads classified to the *Coronavirinae* family, with the positive control spike of Hazara virus detected in 49/53 samples. The percentage of reads classified in total in each sample ranged from 28 to 96% and in both the extraction and library preparation negative controls, no other significant level of reads classified to RNA viruses. Hazara virus was detected in the extraction negative, but not in the library preparation negative.

**Figure 1:**
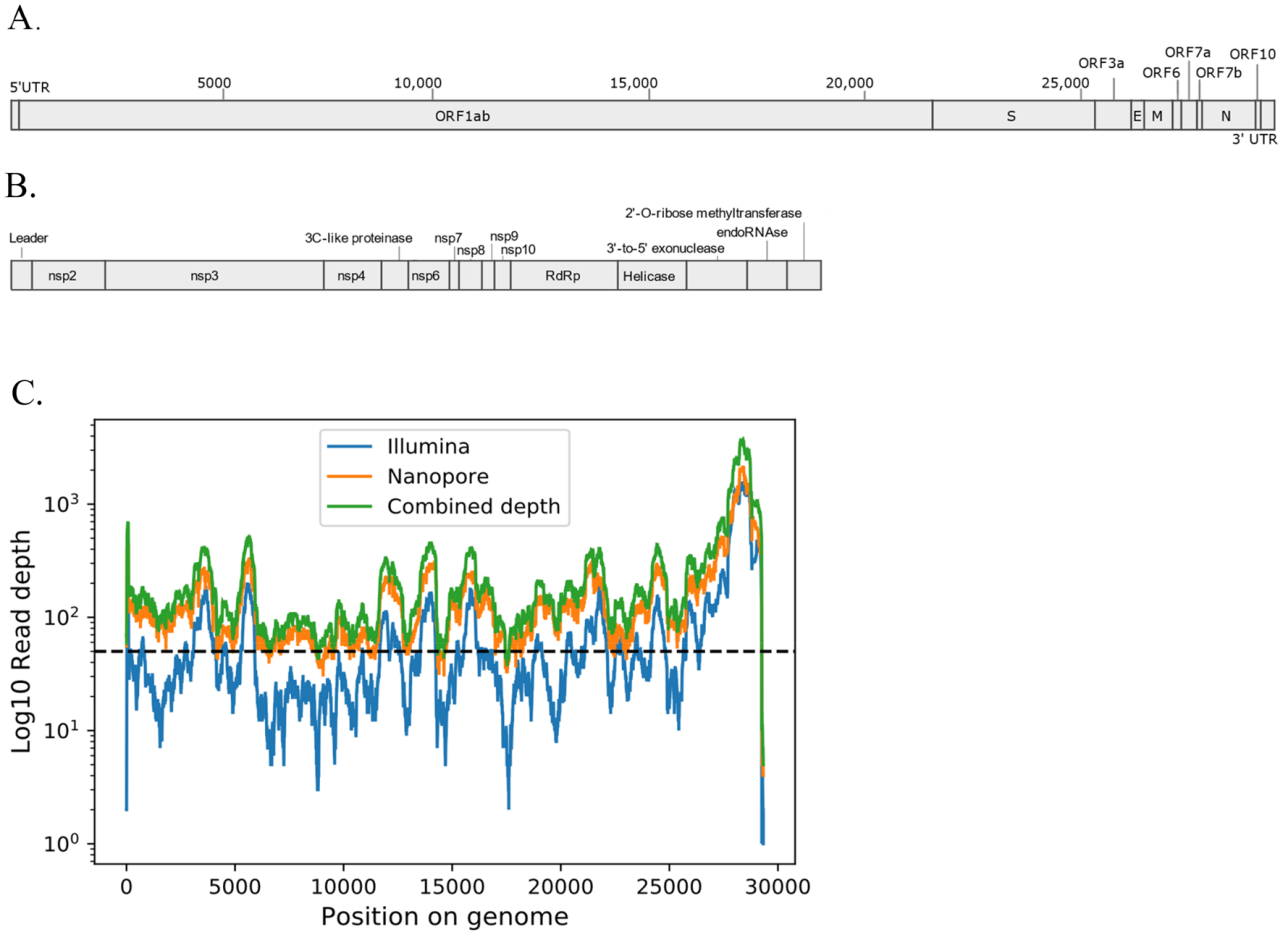
The genomic structure of RhGB01 within the (**A**) entire genome, and (**B**) non-structural proteins. (**C**) Read depth across the genome of RhGB01. Read depth is shown per base across the entire genome from the alignment of Illumina (blue), Nanopore (orange) and combined raw reads (green). The lengths of the genomic features in RhGB01 are 5′UTR (279 bp), ORF1ab (21 kb), S (3.7 kb), ORF3a (813 bp), E (231 bp), M (669 bp), ORF6 (189 bp), ORF7ab (465 bp), N (1.2 kb), ORF10 (78 bp). Total genome length is 29,324 bp.

Classification of the reads in the positive sample identified 0.41% of reads (2550/614,996) as being viral in origin. Of these viral reads, 68% of reads (1668) were classified at species level to bat betacoronavirus BM48-31/BGR/2008 (GenBank reference NC_014470.1)^32^. *De novo* assembly of the Illumina reads generated multiple contigs with homology to members of the subgenus *Sarbecovirus* as assessed via BLASTn; the largest single contig being ~ 7 kb. To investigate, further additional sequence data were generated using Oxford nanopore technology. Using the 562,461 nanopore reads with an average length of ~ 600 kb as scaffold, a hybrid assembly generated a single 29 kb contig similar to sarbecovirus genomes in both size and gene organisation (Fig. 1A,B). To increase confidence in the assembly we performed further Illumina sequencing. With the increased depth, Illumina data alone were assembled into a contig of 21 kb, which was again further assembled to a 29 kb contig with the inclusion of the nanopore data in a hybrid assembly. Mapping all raw reads to this contig shows that 0.97% and 2.37% map to the contig for Illumina and Nanopore respectively. In total, mean read depth along the assembly is ~ 25 × for Illumina data and 20 × for nanopore data. Combined depth coverage across the assembly is ~ 50 ×, confidently supporting the presence of this virus, in the positive sample (Fig. 1C).

BLASTn analysis of the GenBank nr/nt database shows the assembly shares the highest nucleotide identity with a bat betacoronavirus, BtRs-betaCoV/Hub2013 (KJ473814.1), with 81.01% identity across 85% of the assembly. By comparison it shares 79.78% nucleotide identity across 85% of the assembly with SARS-CoV-2 (isolate SARS-CoV-2/human/USA/FL-CDC-STM-000005640/2021, MW586221.1). We named the virus identified as RhGB01 (*Rhinolophus hipposideros*, Great Britain 01) representing the first detection of a sarbecovirus from *R*. *hipposideros* in Great Britain.

**Figure 2:**
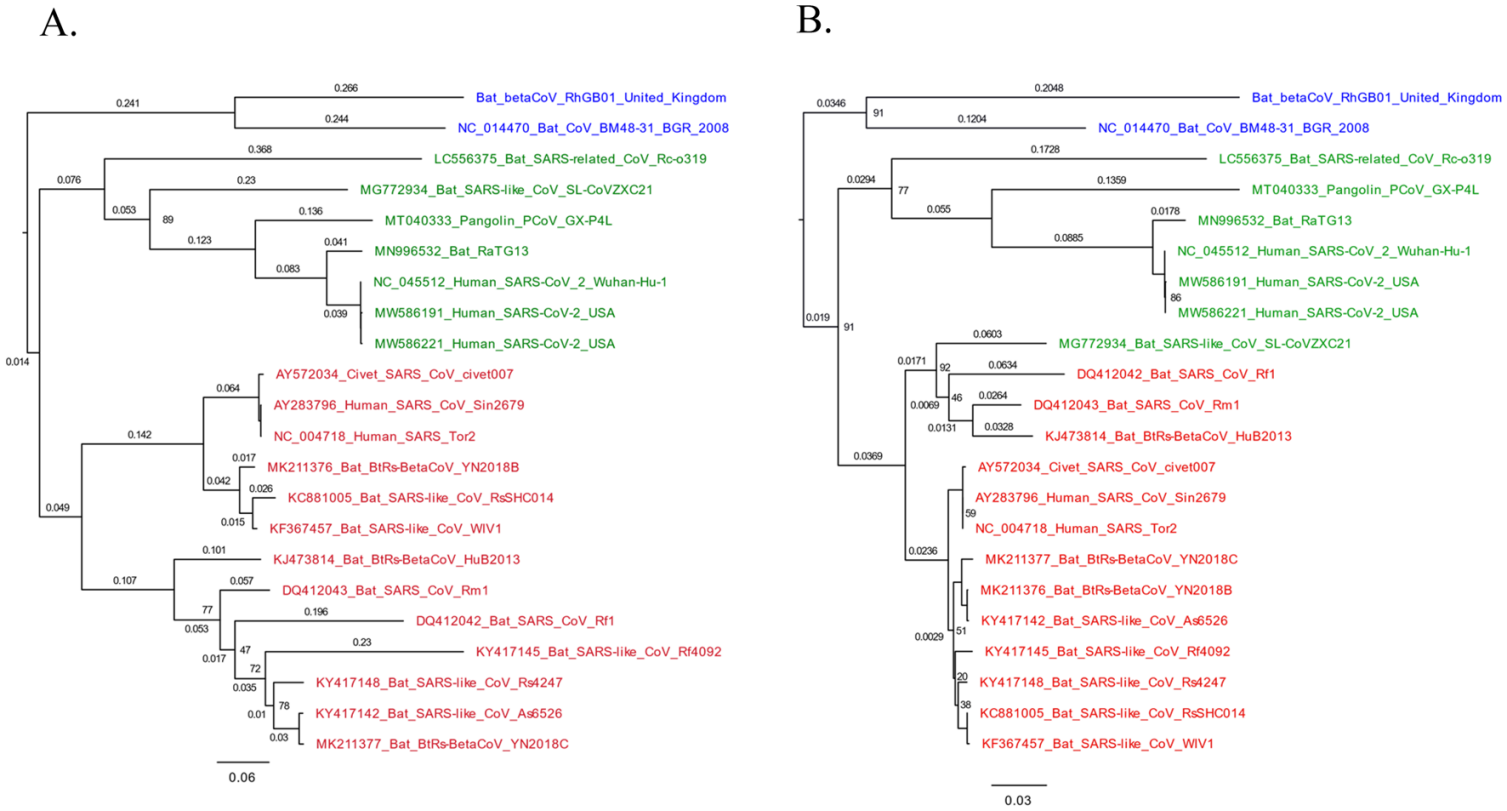
Maximum likelihood phylogenies of the nucleotide sequences for the (**A**) spike glycoprotein and (**B**) RdRp in SARS-CoV, SARS-CoV-2, and related viruses with 1000 bootstrap approximations. Each phylogeny is midpoint rooted and, for visualisation purposes, branch lengths (substitutions per site) in closely related clades and bootstrap supports ≥ 95 are removed. Green taxa are SARS-CoV-2 and related viruses, red taxa are SARS-CoV and related viruses, blue taxa are RhGB01 and BM48-31/GBR-2008.

The genomic structure of RhGB01 mostly mirrors that of other sarbecoviruses with genes encoding non-structural proteins (nsp) in the 5′ region of the genome housed within ORF1ab, and genes encoding structural and accessory proteins at the 3′ region of the genome (Fig. 1A,B)^33^. RhGB01 contains 10 coding genes, whereas SARS-CoV-2, SARS and SARSr-CoV-2 viruses contain 11 coding genes, with the addition of ORF8. In RhGB01, ORF8 and 20 bases in the 5′ region of the ORF7b transcript are absent, comparable to BM48-31/BGR/2008, the closest related virus as determined by phylogenetic analysis (Fig. 2; Supplementary Fig. 1).

### Phylogenetic analysis

Maximum likelihood phylogenies of the spike glycoprotein and RdRp (nsp12) nucleotide sequences demonstrate that RhGB01 clusters in a monophyletic clade with BM48-31/BGR/2008, a sarbecovirus isolated from a Blasius’s horseshoe bat (*Rhinolophus blasii*) in 2008 in Bulgaria (Fig. 2). This clustering is maintained within phylogenies inferred from nucleotide sequences for all structural and accessory proteins (Supplementary Fig. 1a) and most non-structural proteins (Supplementary Fig. 1b). Phylogenies inferred from the 3C-like proteinase, nsp9, helicase and endoRNAse nucleotide sequences demonstrate alterations in the clustering of RhGB01 into the SARS-CoV clade, into a clade with Rc-o139 and into its own clade, respectively (Supplementary Fig. 1b). In all phylogenies, RhGB01 is distinct from clades containing the human pathogenic betacoronaviruses SARS-CoV and SARS-CoV-2 but, of these, is more closely related to SARSr-CoVs.

### The predicted structure of the receptor binding domain of RhGB01 demonstrates structural differences at key residues compared to SARS-CoV and SARS-CoV-2

**Figure 3:**
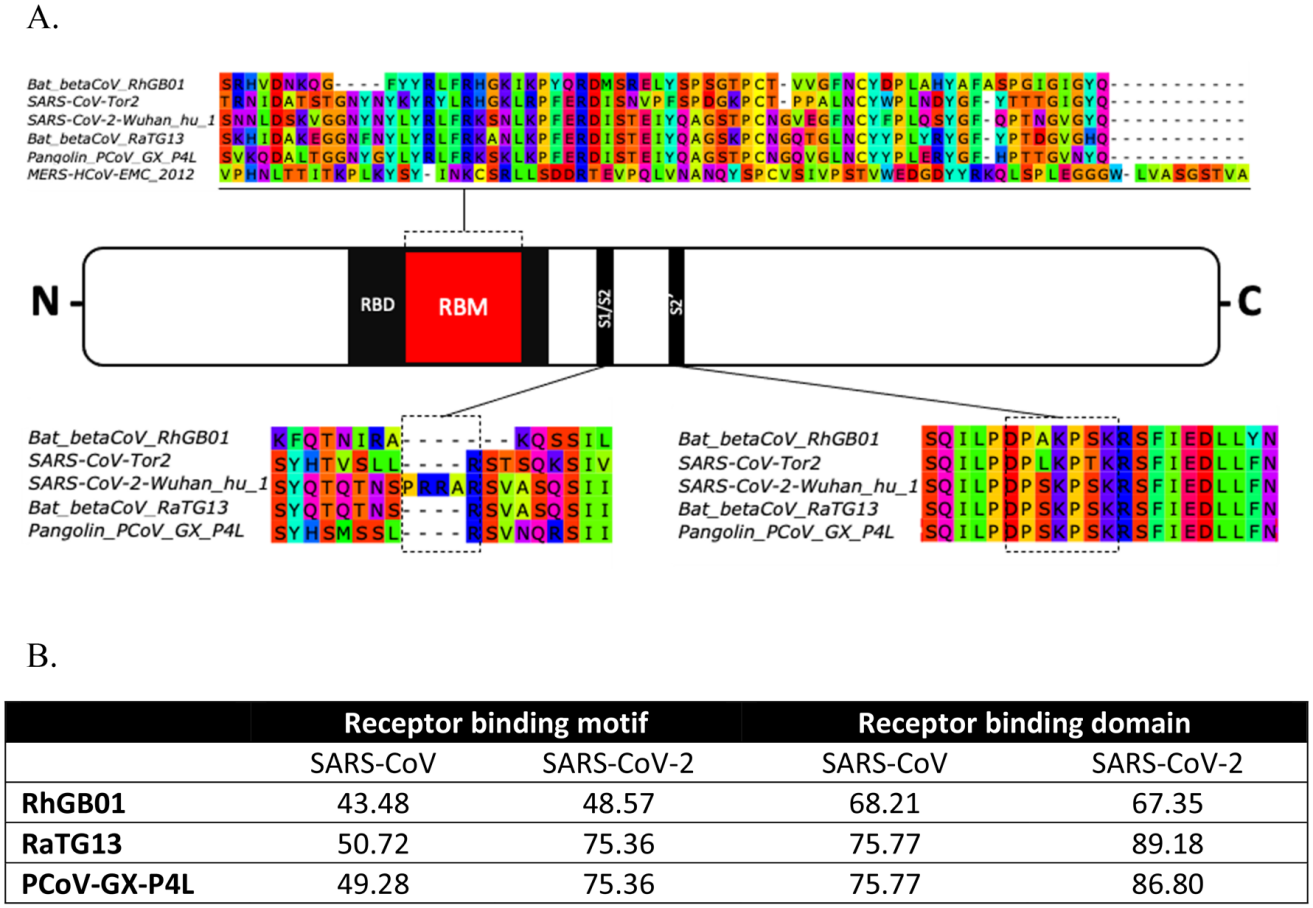
A schematic representation of the entire S protein with the RBM and cleavage sites highlighted and compared between RhGB01, Pangolin and Bat derived related viruses, SARS-CoV and SARS-CoV-2 and percentage identity values for the RBD and RBM regions. Amino acids are coloured according to the Taylor colour scheme. (**A**) RBM comparison demonstrates higher percentages of homology within sarbecoviruses from other zoonotic hosts. The RBM of MERS-HCoV-EMC-2012, subgenus *Merbecovirus,* demonstrates little amino acid homology to RBM from the *Sarbecovirus* subgenus. The furin cleavage site (S1/S2) is only present in SARS-CoV-2, distinct from the TMPRSS2 cleavage motif (S2′), which is more conserved within the sarbecoviruses. (**B**) Percentage identity scores for the RBM and RBD to SARS-CoV and SARS-CoV-2. RaTG13 and PCoV represent the most closely SARSr-CoV-2 virus from zoonotic hosts.

The major human cellular entry receptor for both SARS-CoV and SARS-CoV-2 is Angiotensin-converting enzyme 2 (hACE2). This binding ability is conferred by a receptor binding motif (RBM) within the RBD of the spike glycoprotein. RhGB01 shares amino acid identity of 68% and 67% across the RBD with SARS-CoV and SARS-CoV-2 respectively and just 43% and 48% within the RBM (Fig. 3A). By comparison across the RBD, the closest SARSr-CoV-2 viruses from bat and pangolin hosts share 89% and 86% amino acid homology to SARS-CoV-2 and 75.77% to SARS-CoV (Supplementary Fig. 2). Within the RBM SARSr-CoV-2 viruses from bat and pangolin hosts both share 75% amino acid homology to SARS-CoV-2 and 50% and 49% to SARS-CoV (Fig. 3B). RhGB01 also shows little homology to the RBM of Middle East Respiratory Syndrome (MERS) virus (Fig. 3A). The RhGB01 spike amino acid sequence contains motifs comparable to host transmembrane serine protease 2 cleavage site (TMPRSS2) seen in both SARS-CoV-2 and SARS-CoV in the S2’ target site but lacks the additional furin cleavage site specific to SARS-CoV-2 at the S1/S2 intersection (Fig. 3B).

### Homology modelling

The predicted 3D structure of the RBD contains one α-helix, four 3_10_ helices, three β-bridges and three β-pleated sheets (Supplementary Fig. 3a). It is important to note that the majority of experimentally validated spike glycoprotein and RBD structures are biased towards pathogenic viruses in complex with hACE2 or neutralising antibodies and the accuracy could be improved if the database included more zoonotic spike protein structures.

The predicted 3D structure was validated using ERRAT2 and a Ramachandran plot. The overall quality factor predicted by ERRAT2 is 88.09, supporting the quality of the model. The residues rejected at the 95% and 99% confidence levels are characterised in the literature as contact residues for SARS-CoV to hACE2, highlighting differences within this region compared to the SARS-CoV RBD structure (Supplementary Fig. 3b). A Ramachandran plot predicted that 86% of the residues were in the most favourable region, 13.4% in additional allowed regions and 0.6% in generously allowed regions (Supplementary Fig. 3c).

**Figure 4:**
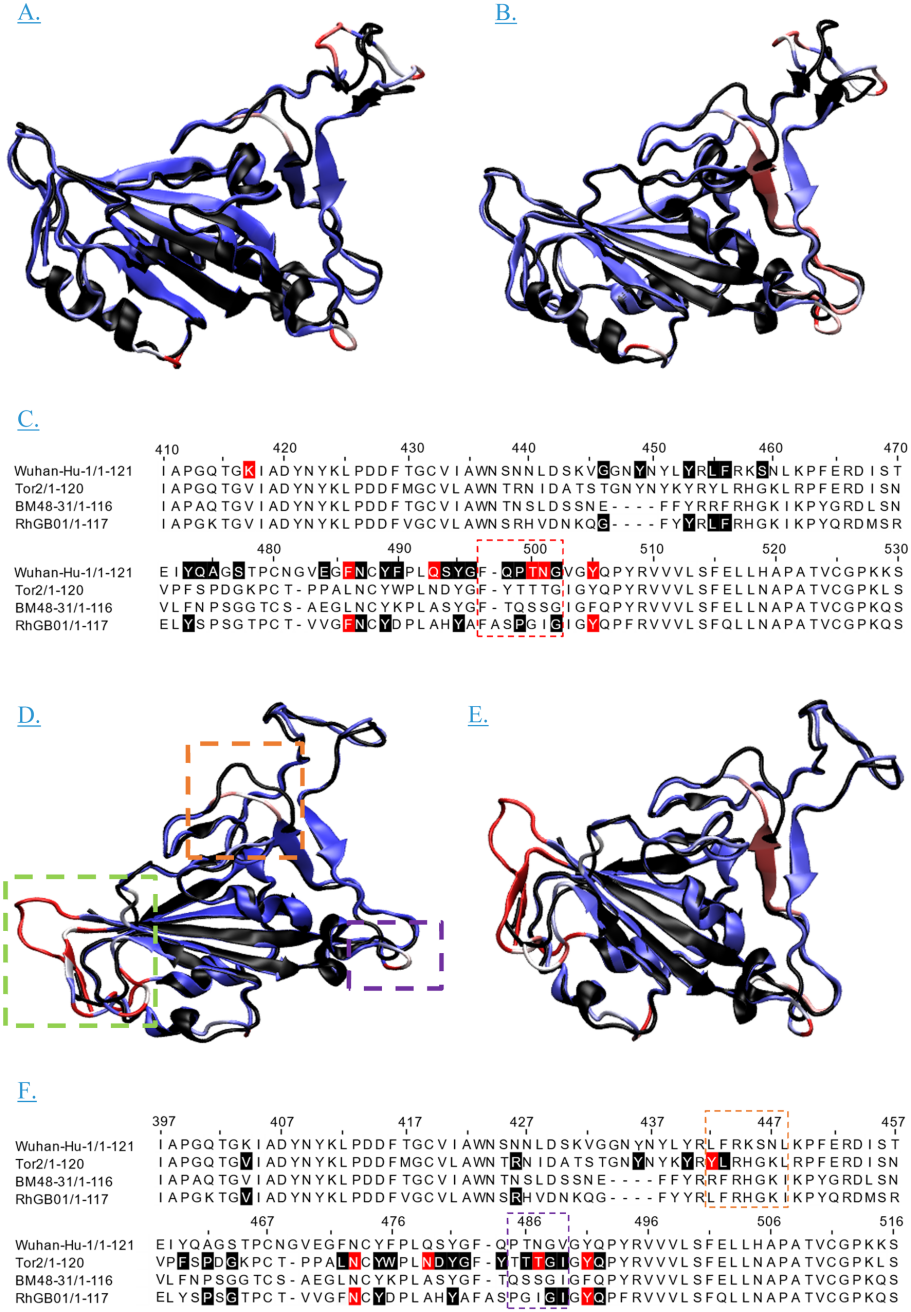
Amino acid sequence and STAMP structural alignments of the RBD of SARS-CoV and SARS-CoV-2 compared to a predicted 3D structure of the RBD of RhGB01 highlighting structural differences in regions housing hACE2 contact residues for SARS-CoV and SARS-CoV-2. Contact residues are described in the literature (black), with residues in red the most identified residues. Regions coloured in purple have high structural similarity (> 0.7) and residues with a low structural similarity coloured in red/white (< 0.3). BM48-31 is included as comparison to a second Sarbecovirus from a bat source. (**A**) STAMP alignment of SARS-CoV-2 RBD (PDB ID 6M0J) in the conformation of being complexed with hACE2 aligned with the RhGB01 RBD. The areas where structural similarity is low are seen to contain contact residues defined in the literature. (**B**) Sequence alignment of RhGB01 and SARS-CoV-2. The sequence used is identical to (**C**). (**C**) Sequence alignment of the RBM with 28 residues up and downstream, to facilitate the inclusion of K417. The coordinates provided refer the SARS-CoV-2 (NC_45512.2) residue position within the spike protein alignment. (**D**) STAMP alignment of SARS-CoV RBD (PBD ID 2DD8) aligned with the predicted RhGB01 RBD. The region highlighted in green are residues upstream and downstream of the structural alignment which do not align. (**E**) Sequence alignment of RhGB01 and SARS-CoV. (**F**) Sequence alignment of RBM with SARS-CoV contact residues highlighted, and the coordinates provided refer to the residue position for SARS-CoV (NC_004718.3) within the spike protein alignment. 28 residues are included up and downstream of the RBM to include V404.

Structural conservation within the RBD of sarbecoviruses, including RhGB01, is predicted as the RBD is required for viral entry into host cells via the ACE2 receptor. The predicted RBD structure of RhGB01 is more conserved to both SARS-CoV and SARS-CoV-2 than sequence alignment alone indicates, but still differs in regions containing key contact residues for binding hACE2^34–42^ (Fig. 4).

## Discussion

Here we discovered a novel sarbecovirus (RhGB01), the first to be described in the UK, after sampling just 53 lesser horseshoe bat faecal samples. It is possible that the infection prevalence was even higher and RT-PCR, which would improve sensitivity of detection, should be utilised to screen British bat samples in the future now a sarbecovirus has been detected in the UK. While other sarbecoviruses have been identified in rhinolophid bats in other European countries by polymerase chain reaction and partial gene sequence analyses, RhGB01 is only the second from Europe to be fully sequenced^43^, and the first from a lesser horseshoe bat. The only other full sequence betacoronavirus from a European horseshoe bat is BM48-31/BGR/2008 from *R. blasii*. Our results, therefore, extend the geographic and species ranges of SARSr-CoVs and suggest that sarbecoviruses are likely to be present throughout the range of the Rhinolophidae, which are distributed from Australia and Japan to Europe and Africa.

The range of the lesser horseshoe bat extends from Western Europe to Central Asia, overlapping with those of other rhinolophid species, including the greater horseshoe bat (*R. ferrumequinum*), which ranges from Western Europe to Japan^44,45^. Where they co-exist, the species can be syntopic allowing opportunity for cross-species virus transfer. Prior to our results, the observed and predicted (cut off ≥ 0.9821) number of coronaviruses in the greater horseshoe bat were 13 and 19 respectively, and in the lesser horseshoe bat these figures were zero and three respectively^46^. This suggests that the complement of *Sarbecovirus* species in horseshoe bats is greater than predicted so far, with the possibility of virus sharing across species and large geographic areas.

Genomic alignments between RhGB01 and related sarbecoviruses highlight key genomic differences between RhGB01 and known zoonotic sarbecoviruses. Host specificity is dependent on the ability of a virus to attach to host receptors and enter host cells; a binding process facilitated by contact residues contained within the RBM^37^. RhGB01 demonstrates low amino acid homology to SARS-CoV and SARS-CoV-2 in the RBM compared to that between SARS-CoV, SARS-CoV-2 and the closely related bat (RaTG13) and pangolin (PCoV_GX_P4L) sequences identified in Asia^48,49^. The low level of homology, lack of contact residues and structural differences compared to the RBD of SARS-CoV and SARS-CoV-2 most likely indicate a lack of ability to bind hACE2 and, hence, RhGB01 is unlikely to be zoonotic without mutation. To experimentally validate the absence of hACE2 or other human cell receptor binding and to identify binding abilities to other mammalian ACE2 receptors, in vitro binding assays are required.

Aside from the variation observed in amino acid homology within the RBM, RhGB01 also exhibits variation within the furin cleavage site and ORF8 when compared to zoonotic sarbecoviruses. The absence of the furin cleavage site indicates the absence of enhanced efficiency of host cell entry observed with SARS-CoV-2^50^. However, RhGB01 does retain a similar motif responsible for cleavage in the S2’ region by host transmembrane serine protease 2, also required for spike protein proteolytic priming for ACE2 attachment^47^. SARS-CoV-2 variants with a functional ORF8 are associated with greater pathogenicity, thought to be due to downregulation of major histocompatibility complex class 1 (MHC 1), and thus a reduction in antigen presentation to CD8^+^ T lymphocytes which facilitates prolonged infection^51,52^. The absence of ORF8 from the genome of RhGB01 suggests that this virus lacks these immune evasion properties.

It has been postulated that both SARS-CoV and SARS-CoV-2 evolved through mutation, possibly involving homologous recombination, during passage through at least one intermediate host; probably civets (viverrids) or mustelids for SARS-CoV^53,54^ and possibly a species of pangolin for SARS-CoV-2^49^. In this way, the progenitor virus from the natural host (a species of horseshoe bat) gained genetic adaptations to allow successful infection of, and transmission between, human beings. Where there is opportunity for homologous recombination of sarbecoviruses through co-infection, there is the possibility of novel zoonotic emergence. Thus, co-infection of horseshoe bats with their natural suites of coronaviruses and with SARS-CoV-2 could lead to the development of novel zoonotic emergence. While there is a need to increase surveillance for coronaviruses in horseshoe bats across their range, and also in other bat species, especially those syntopic with, or closely related to, horseshoe bats (e.g. the Old World leaf-nosed bats, family Hipposideridae), it is also important that steps are taken to minimise opportunities of virus transmission between novel hosts.

In Europe, unlike in Asia, direct contact between people and bats most commonly occurs when the animals are captured by bat researchers or when sick animals are taken in by bat rescuers and wildlife rehabilitation centres. While the risk of reverse spill over of SARS-CoV-2 from researchers to bats and onward spread within bat populations has been shown to be medium to high^55^, it is the caring of sick or injured bats, in particular, that provides most opportunity for long-term close contact and virus transfer in either direction. Although the IUCN Bat Specialist Group has produced guidelines to minimise this risk^56^, the degree to which these are known or followed is unclear. Our findings highlight that the natural distribution of sarbecoviruses and opportunities for recombination through intermediate host co-infection are underestimated. Preventing transmission of SARS-CoV-2 to horseshoe bats, with the risk this presents of further mutation, is of particular significance with the current roll out of a global mass vaccination campaign against this virus.

## Supplementary Information

Supplementary Figure S1.

Supplementary Figure S2.

Supplementary Figure S3.
