# Supplementary material for: Metagenomic identification of a new sarbecovirus from horseshoe bats in Europe
Source: Sci Rep. 2021 Jul 19;11:14723. doi: 10.1038/s41598-021-94011-z (PMC8289822; doi:10.1038/s41598-021-94011-z)
Supplement: Supplementary file 1 — Supplementary Figure S1. [file 41598_2021_94011_MOESM1_ESM.docx]

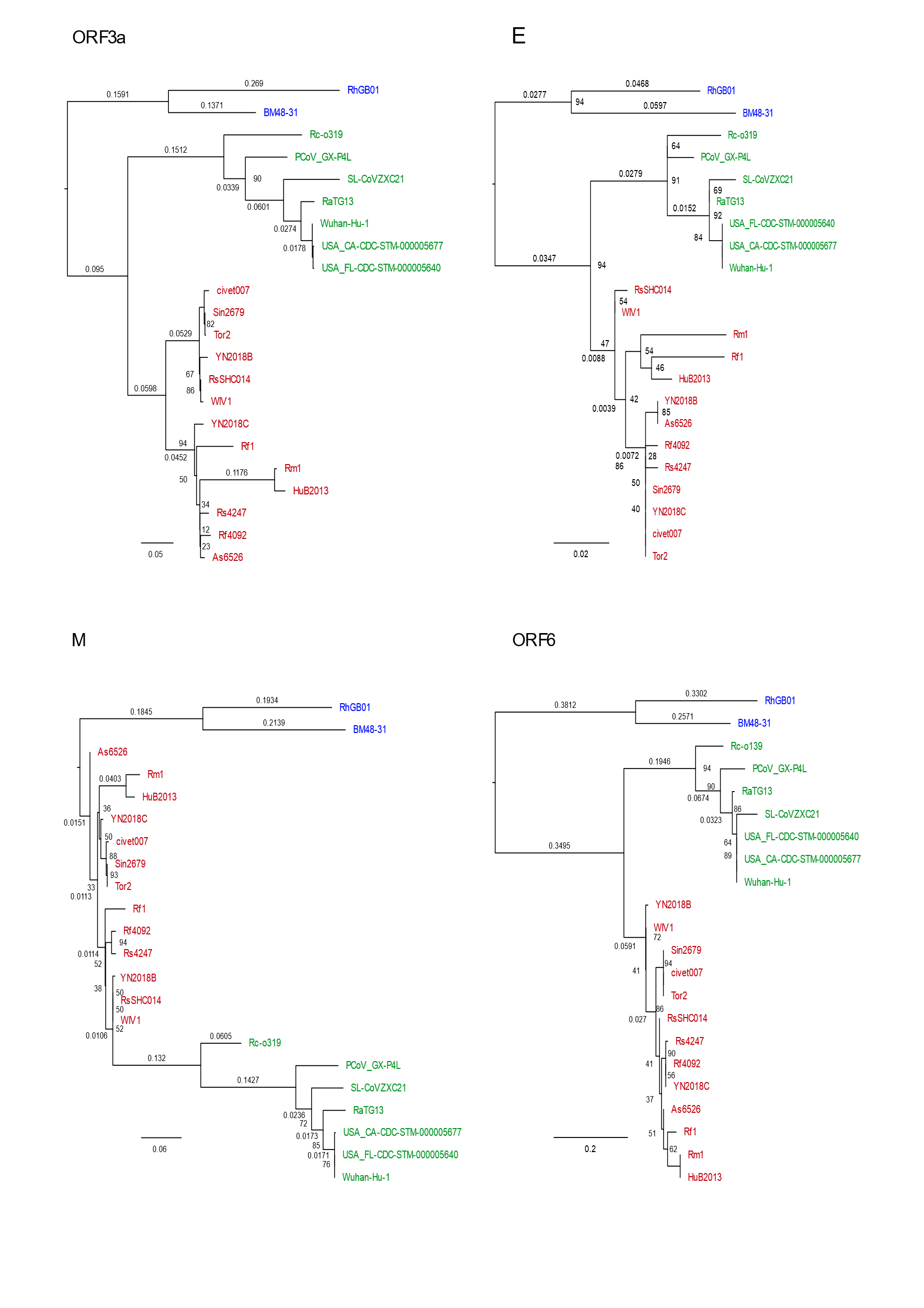


A.

ORF3a

E

M

ORF6


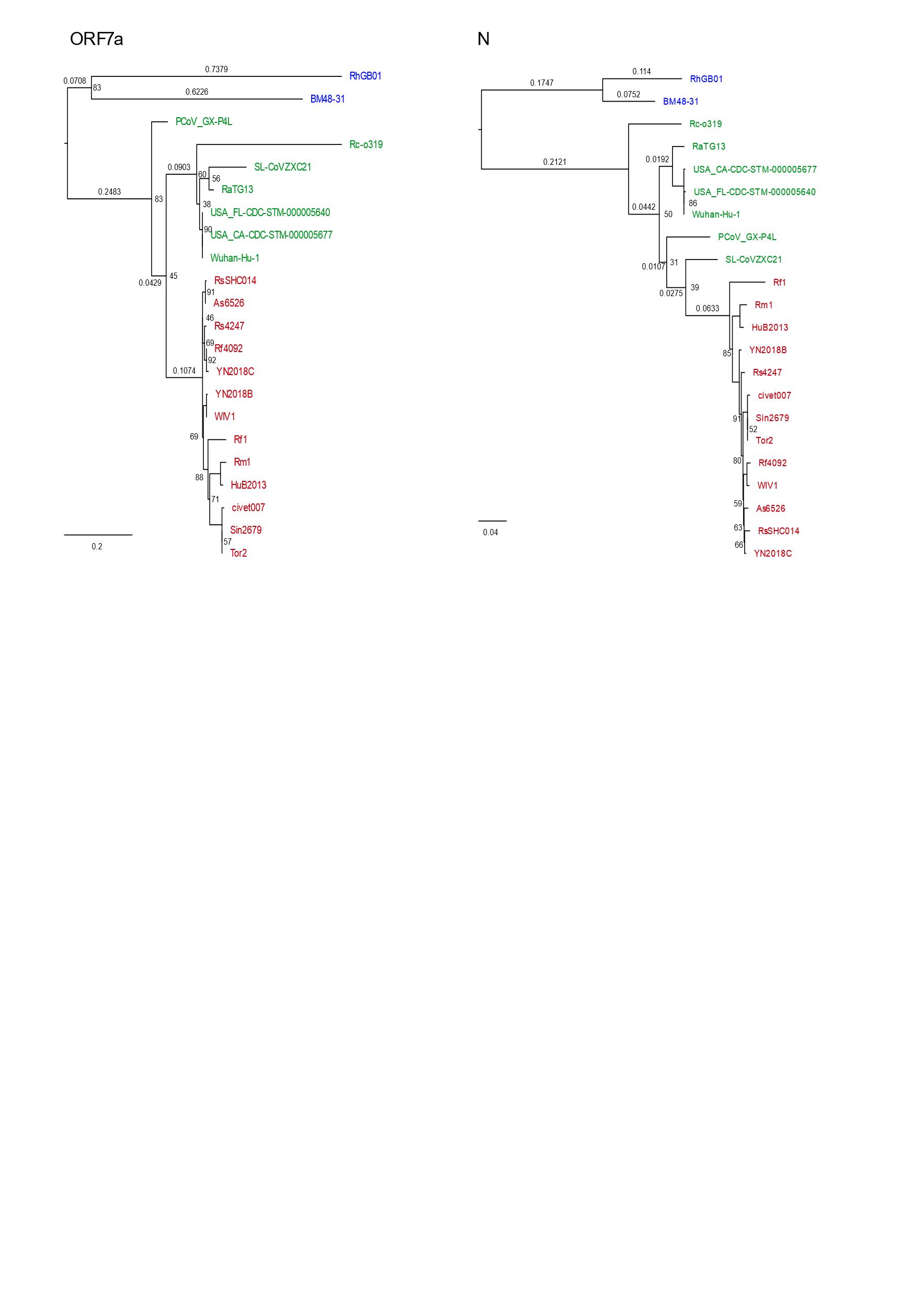


ORF7a

N


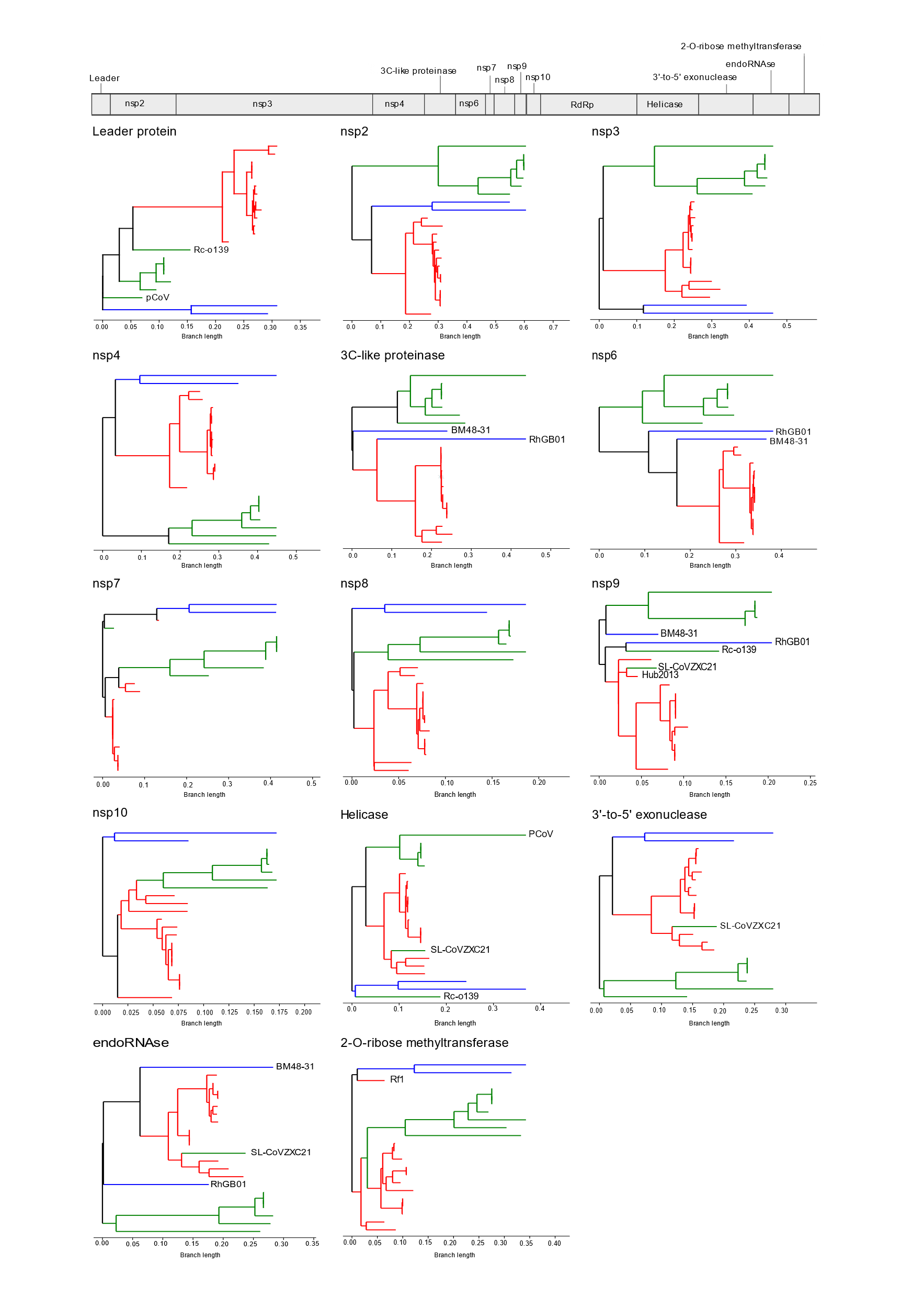


B.

Leader protein

nsp2

nsp3

nsp4

3C-like proteinase

nsp6

**Supplementary figure 1.**

Maximum likelihood phylogenies from nucleotide sequences of A) structural and accessory proteins and B) non-structural proteins. All phylogenies are midpoint rooted and for visualisation purposes, bootstrap support values >95 and branch lengths on closely related terminal branches are removed. Scales provided are substitutions per site per year. In all phylogenies, taxa in blue represent RhGB01 and BM48-31/BGR/2008, green are SARS-CoV and related and red are SARS-CoV-2 and related. ORF7b and ORF10 are omitted as the sequence is not long enough for meaningful phylogenetic inference.


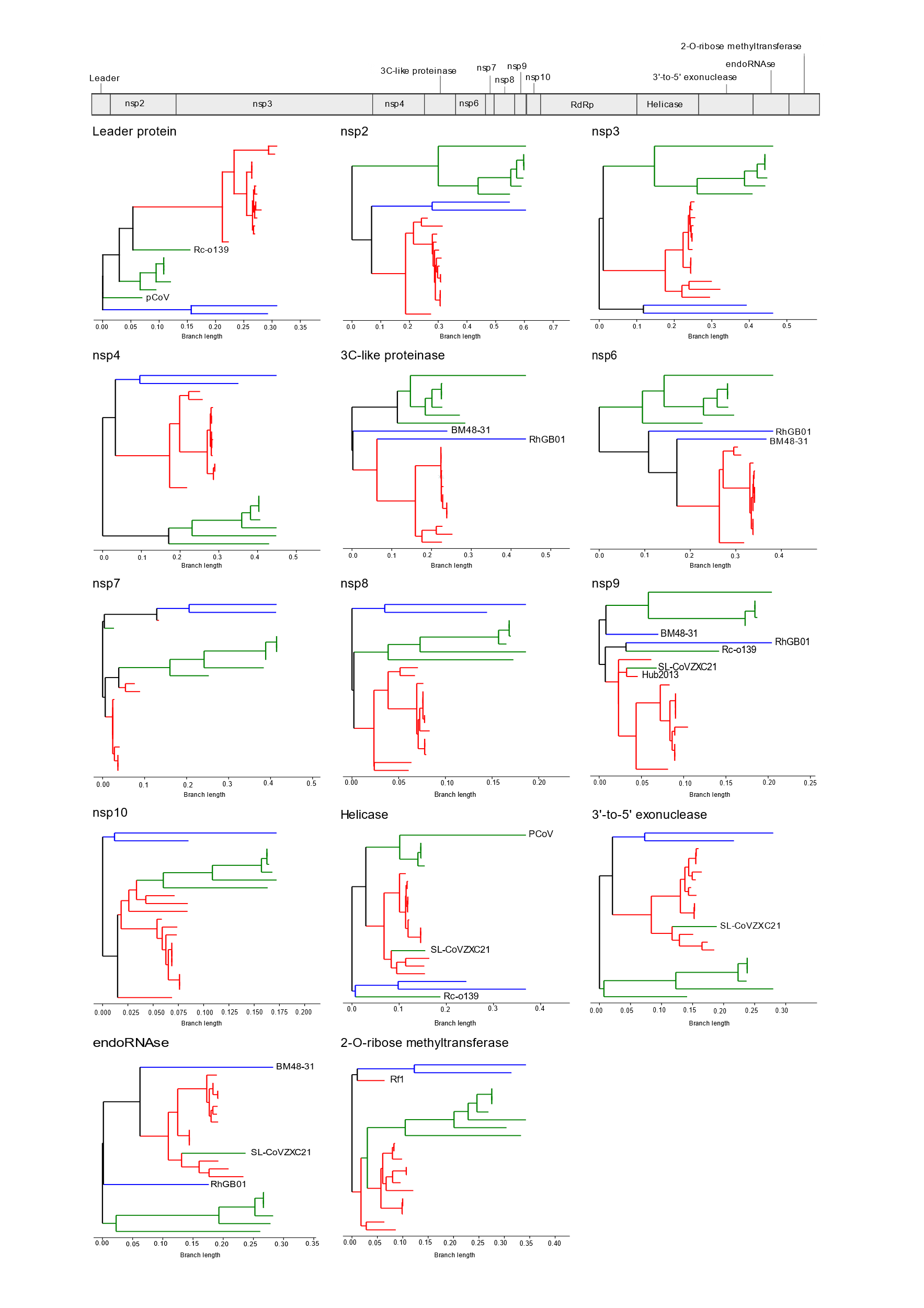


nsp7

nsp8

nsp9

nsp10

Helicase

3’-to-5’ exonuclease

EndoRNAse

2’-O-ribose methyltransferase
