# Supplementary material for: Metagenomic identification of a new sarbecovirus from horseshoe bats in Europe
Source: Sci Rep. 2021 Jul 19;11:14723. doi: 10.1038/s41598-021-94011-z (PMC8289822; doi:10.1038/s41598-021-94011-z)
Supplement: Supplementary file 2 — Supplementary Figure S2. [file 41598_2021_94011_MOESM2_ESM.docx]

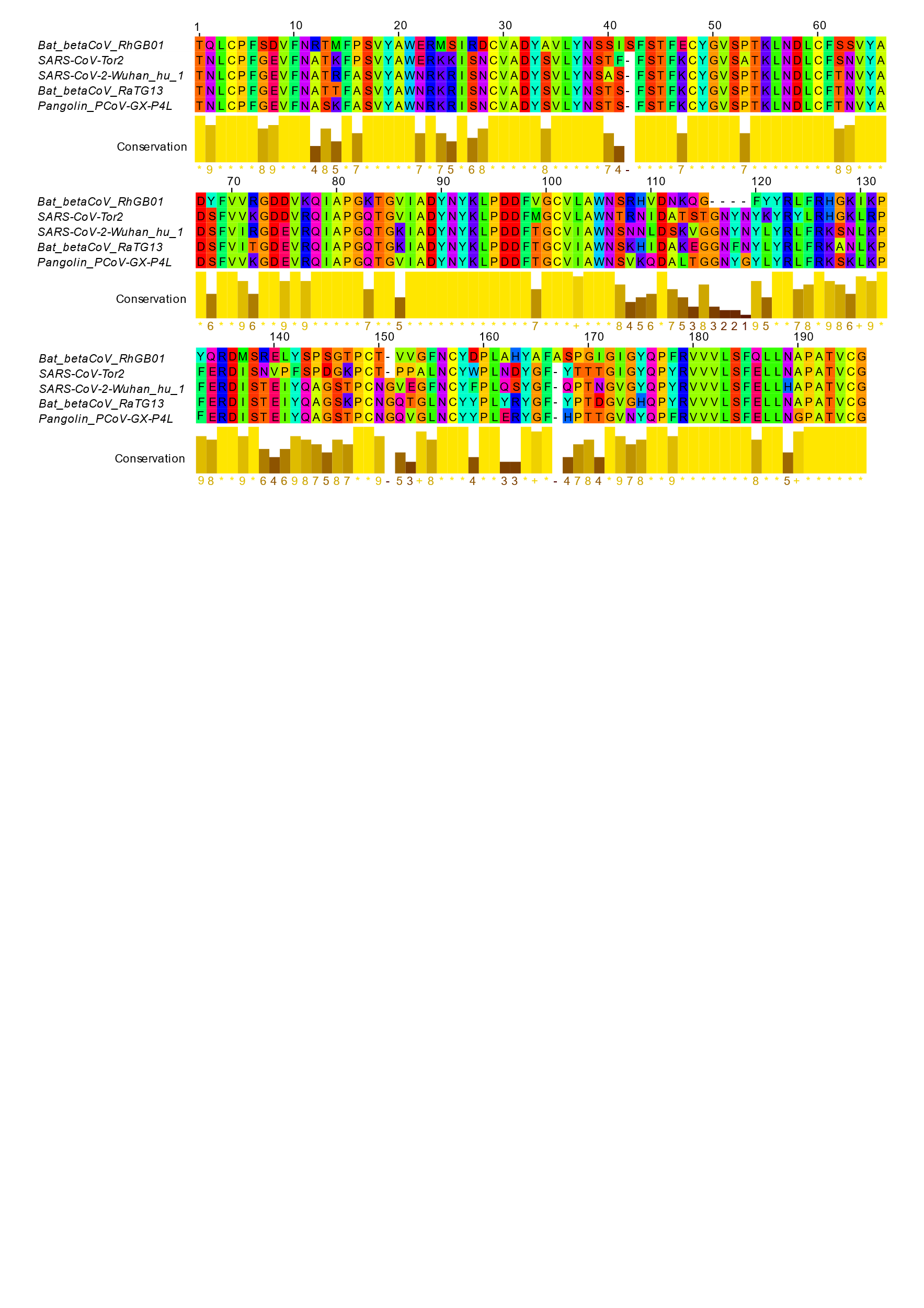


**Supplementary Figure 2.**

Amino acid homology between RhGB01, zoonotic SARSr-CoV, SARS-CoV and SARS-CoV-2 vary between the RBD and RBM. Conservation represents physiochemical properties of amino acid residues at that site.
