# Supplementary material for: Metagenomic identification of a new sarbecovirus from horseshoe bats in Europe
Source: Sci Rep. 2021 Jul 19;11:14723. doi: 10.1038/s41598-021-94011-z (PMC8289822; doi:10.1038/s41598-021-94011-z)
Supplement: Supplementary file 3 — Supplementary Figure S3. [file 41598_2021_94011_MOESM3_ESM.docx]

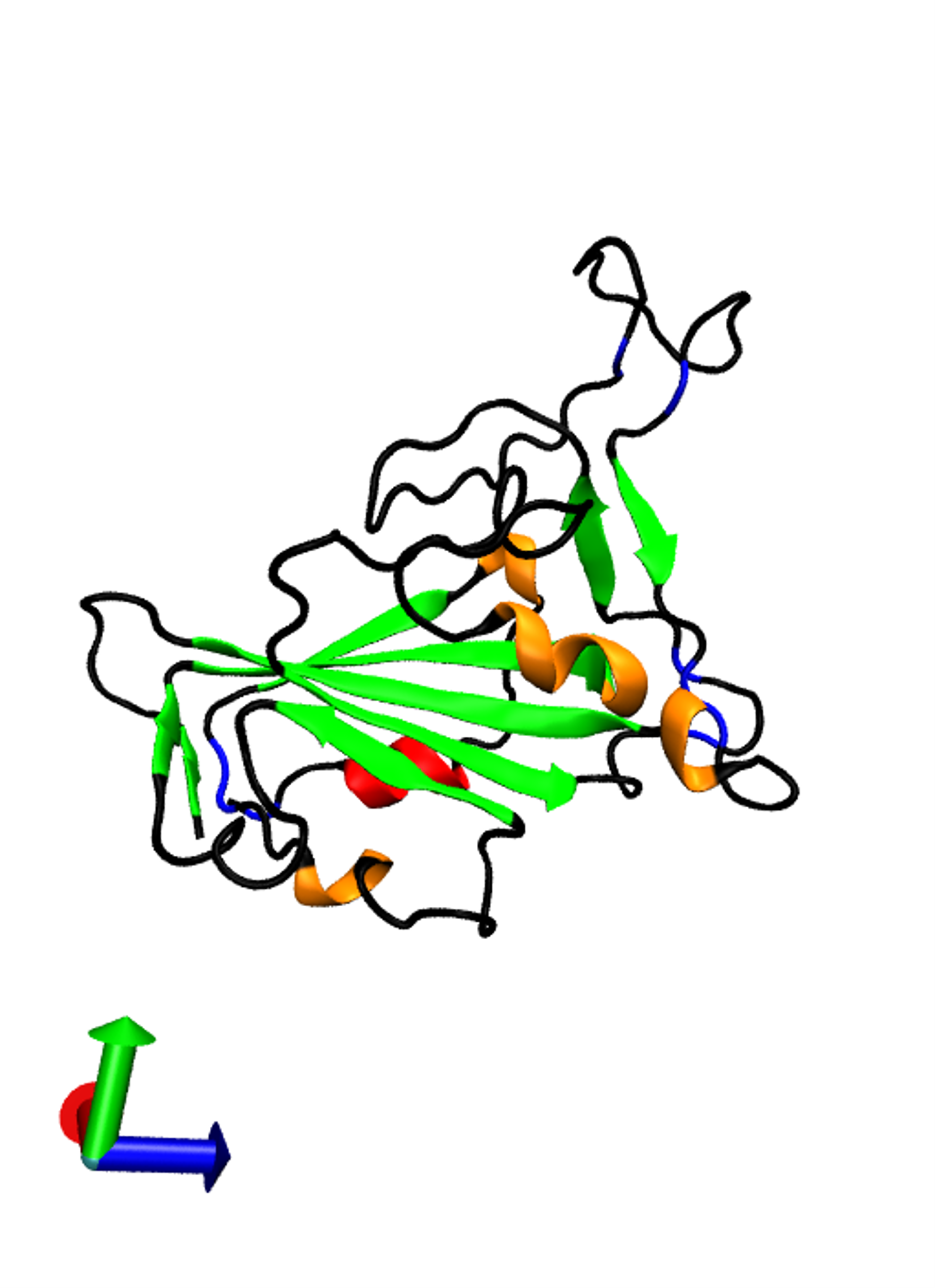

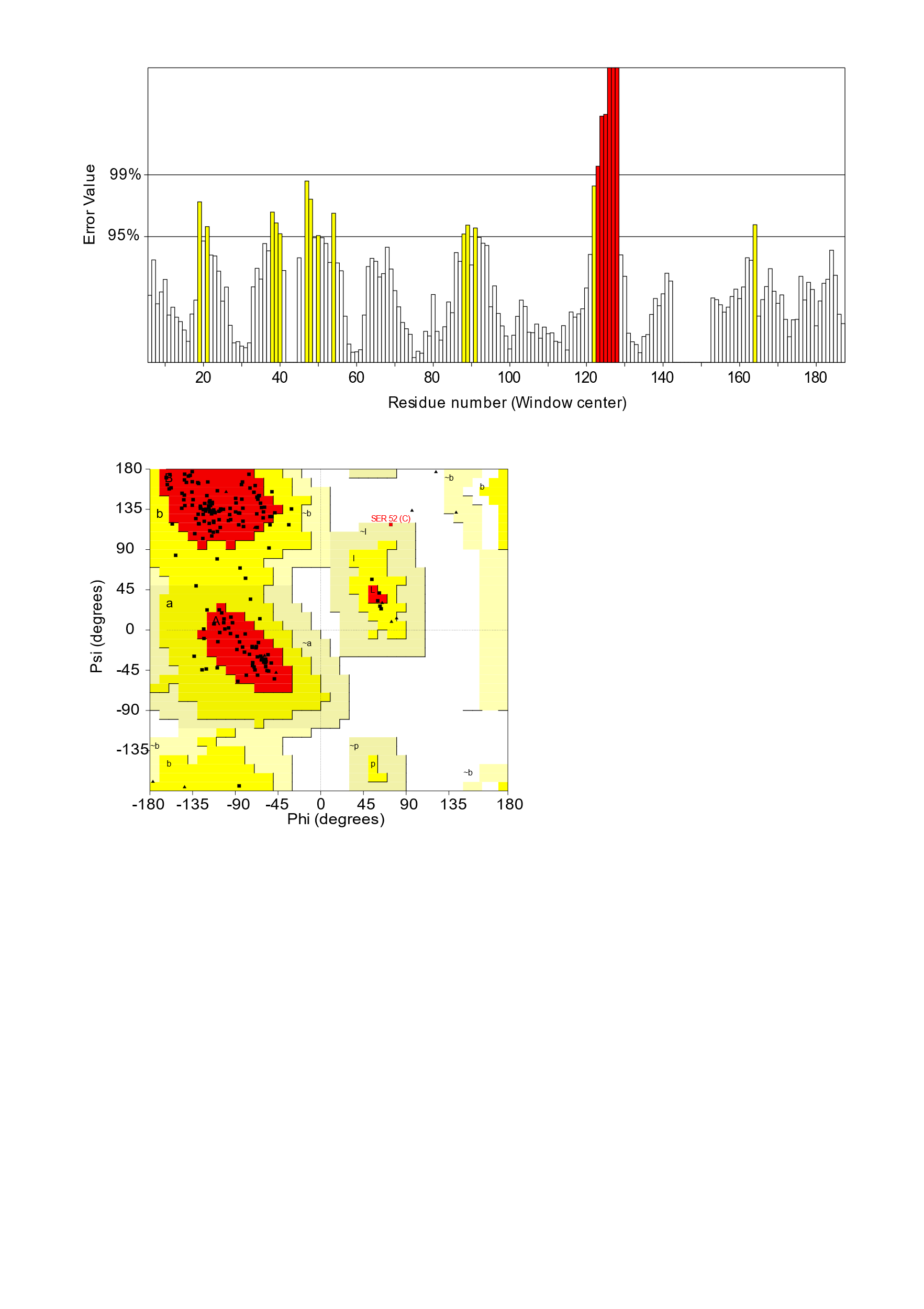

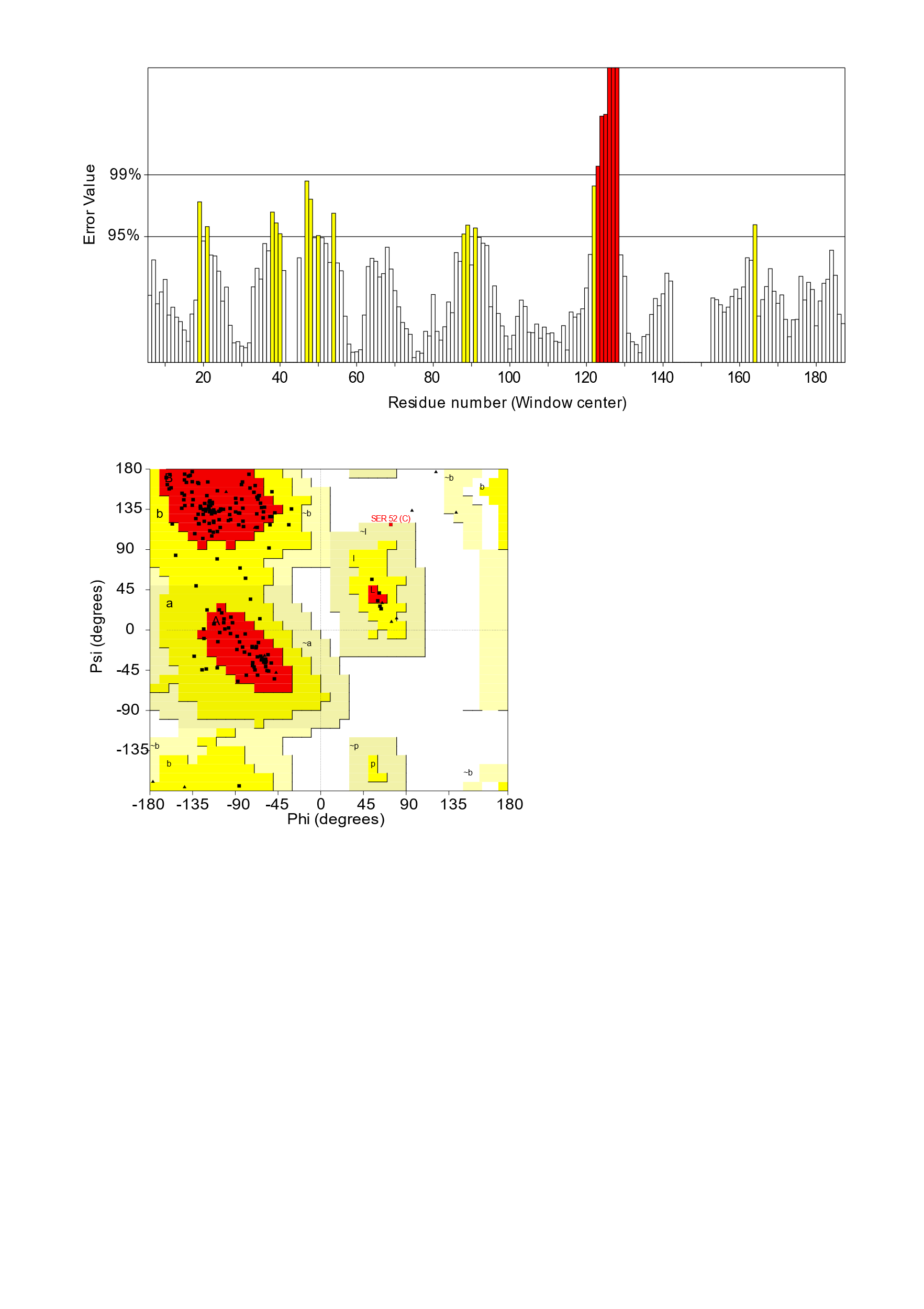


A.

B.

C.

**Supplementary figure 3.**

SWISS-MODEL was used for homology modelling to generate a predicted 3D structure of the RhGB01 RBD amino acid sequence (324-515). A) A visual representation of the RhGB01 RBD with 3_10_ helices in orange, extended β-sheers in green, β-bridge in blue, α-helix in red and turns/coils in black. B) ERRAT2 evaluation of the quality of the model. The y-axis determines the two error lines (95 and 99%) to indicate the confidence of rejection for regions of the predicted structure using a sliding 9-residue window. Bars highlighted in yellow and red surpass the 95% and 99% cut off, respectively. C) A Ramachandran plot analysis which indicates the favoured regions (red), allowed regions (yellow) and generously allowed regions (light yellow).
